# Accessibility to tuberculosis control services and tuberculosis programme performance in southern Ethiopia

**DOI:** 10.3402/gha.v8.29443

**Published:** 2015-11-20

**Authors:** Mesay Hailu Dangisso, Daniel Gemechu Datiko, Bernt Lindtjørn

**Affiliations:** 1 Centre for International Health, Faculty of Medicine and Dentistry, University of Bergen, Bergen, Norway; 2 School of Public and Environmental Health, College of Medicine and Health sciences, Hawassa University, Hawassa, Ethiopia; 3 Sidama Zone Health Department, Hawassa, Ethiopia; 4 HHA – REACH Ethiopia, Hawassa, Ethiopia; 5 Department of Clinical Sciences, Liverpool School of Tropical Medicine, Liverpool, United Kingdom

**Keywords:** public health, population health, tuberculosis control, health systems, developing countries, Africa

## Abstract

**Background:**

Despite the expansion of health services and community-based interventions in Ethiopia, limited evidence exists about the distribution of and access to health facilities and their relationship with the performance of tuberculosis (TB) control programmes. We aim to assess the geographical distribution of and physical accessibility to TB control services and their relationship with TB case notification rates (CNRs) and treatment outcome in the Sidama Zone, southern Ethiopia.

**Design:**

We carried out an ecological study to assess physical accessibility to TB control facilities and the association of physical accessibility with TB CNRs and treatment outcome. We collected smear-positive pulmonary TB (PTB) cases treated during 2003–2012 from unit TB registers and TB service data such as availability of basic supplies for TB control and geographic locations of health services. We used ArcGIS 10.2 to measure the distance from each enumeration location to the nearest TB control facilities. A linear regression analysis was employed to assess factors associated with TB CNRs and treatment outcome.

**Results:**

Over a decade the health service coverage (the health facility–to-population ratio) increased by 36% and the accessibility to TB control facilities also improved. Thus, the mean distance from TB control services was 7.6 km in 2003 (ranging from 1.8 to 25.5 km) between *kebeles* (the smallest administrative units) and had decreased to 3.2 km in 2012 (ranging from 1.5 to 12.4 km). In multivariate linear regression, as distance from TB diagnostic facilities (b-estimate=−0.25, *p<*0.001) and altitude (b-estimate=−0.31, *p*<0.001) increased, the CNRs of TB decreased, whereas a higher population density was associated with increased TB CNRs. Similarly, distance to TB control facilities (b-estimate=−0.27, *p*<0.001) and altitude (b-estimate=−0.30, *p*<0.001) were inversely associated with treatment success (proportion of treatment completed or cured cases).

**Conclusions:**

Accessibility to TB control services improved despite the geographic variations. TB CNRs were higher in areas where people had better access to diagnostic and treatment centres. Community-based interventions also played an important role for the increased CNRs in most areas.

Health service coverage (HSC) and access are commonly used indicators in health policy planning and management. Different studies describe the HSC (‘potential coverage’) as the ability of a healthcare facility to provide services for a target population (1) and categorise access to health services as availability, accessibility, accommodation, affordability, and acceptability (2, 3). The availability or supply of services could precede other dimensions of access; however, the availability of health services per se may not assure their utilisation. In principle, all segments of the population should have standards of equivalent healthcare at all levels (4). However, as is true for other public services, healthcare is not equally distributed and accessible to all individuals or groups in the community. In this study, we focus on the physical accessibility to and availability of tuberculosis (TB) control services.

Spatial accessibility (2, 3), the physical closeness to or distance from a healthcare facility, is one of the factors that affect the utilisation of available health services (5). The use of residential locations to measure physical accessibility to health facilities helps identify the disparities and inequity in health service provision in terms of geographic distribution and accessibility (3, 6). Geographic information systems (GIS) can be used to assess the accessibility to healthcare services (7, 8), healthcare providers (9, 10), and for planning appropriate locations for health facilities (11). Few studies from Africa have also used GIS to assess accessibility to TB control facilities and provided important information for TB control programmes (12, 13).

The Ethiopian healthcare delivery system is divided into three levels: the primary healthcare level (district hospitals, health centres, and health posts); the secondary level, comprising general hospitals; and the tertiary level, consisting of specialised hospitals (14). In Ethiopia, physical access to health services is measured on the basis of 10 km distance to a health facility from residential locations, and the HSC is determined by the health-facilities-to-population ratio (15).

Ethiopia is one of the high TB burden countries implementing the Directly Observed Treatment Short-Course (DOTS) strategy for about two decades, and the country has carried out a substantial expansion of primary healthcare facilities. In Ethiopia, the incidence of TB was 224 per 100,000 people in 2013 (having declined from 342 in 2005). TB case notification rates (CNRs) and treatment outcomes are used to measure the performance of a TB control programme. The CNR is the number of TB cases recorded per 100,000 people for a given year. In 2013, 131,677 cases (140 cases per 100,000 people) of all forms of TB and 43,860 smear-positive pulmonary TB cases were recorded in Ethiopia. In the same year, about 30,000 deaths were reported in HIV-negative TB cases. Following the expansion of DOTS services, the CNRs and treatment outcomes improved. Thus, the proportion of new smear-positive TB cases who were successfully treated (having completed treatment or been cured) increased from 80% in 2000 to 89% in 2011, while the proportions of loss to follow-up and mortality declined (16). Despite the improvement in the performance of TB control programmes, the CNRs vary in different years and between administrative regions within the country (ranging from 56 to 311 per 100,000 people) (16, 17).

Studies from Ethiopia reported that active case finding involving health extension workers (community health workers) improved TB case detection and treatment outcomes (18, 19). The health extension workers carry out promotive, preventive, and basic curative health services. They also carry out identification of TB suspects and referral of the suspects for diagnosis, and conduct treatment follow-up. In 2011, an active case-finding intervention involving health extension workers was launched in the Sidama Zone in southern Ethiopia aimed at improving TB program performance (19). An earlier study from the study area (in the Sidama Zone) reported an increased trend and variations in TB CNRs (20). This increase could be related to variations in the HSC and access in different settings. Studies also report the relationship of high population density and crowding (21–23) with increased TB case notification, and the relationship of higher altitude with lower incidence of TB (21, 24, 25).

Accessibility to TB control services can affect treatment outcomes. Evidence shows that travelling long distances to treatment facilities and inconsistent availability or supply of drugs could contribute to poor treatment success and a higher loss to follow-up (26, 27).

There is no information from Ethiopia that analyses how TB control services are spatially distributed and accessible to the community. Moreover, limited information exists about the relationship between physical accessibility to TB control services and smear-positive pulmonary TB CNRs and treatment outcomes. As a result, we aimed to study the geographical distribution of and physical accessibility to TB control services and their relationship with smear-positive PTB case notification and treatment outcomes.

## Methods

### Study area and setting

This study was conducted in the Sidama Zone, one of the most densely populated areas in Ethiopia, with a population of over 3.4 million (28). The Sidama Zone is divided into 19 districts, 2 town administrations, and 524 rural and 39 urban *kebeles*. Kebeles are the smallest administrative units, containing about 5,000 people on average. Modern healthcare services in the Sidama Zone started almost six decades ago in Yirgalem. Since then the trend in expansion of healthcare facilities was steady until 2010. In the study area, the DOTS services started in 1995 and the number of health facilities providing DOTS increased from 65 in 2003 to 114 in 2012 (Supplementary Table 1). In 2012, the number of health facilities with functional sputum smear microscopy services was 81 (having increased from 26 in 2003).

### Study design

We employed an ecological study design because we aggregated the number of TB cases at the kebele level to compute the CNRs and treatment outcomes. We used the lowest administrative level (the kebele) as a unit of analysis in order to assess the relationship of case notification and treatment outcomes to physical accessibility and environmental variables such as population density and altitude.

### Data collection procedure

The study was carried out from August to September 2012 in all DOTS-providing health facilities. We obtained population data for each kebele and the geographic information of the enumeration locations (ELs) from the Central Statistical Agency of Ethiopia (28). Geographic positioning system (GPS) receivers and a structured questionnaire were used for data collection. The data collectors interviewed the heads of district health offices or persons in charge at health facilities for information about health facility type, year of establishment, ownership, and availability of TB control services (availability of laboratory services, reagents, drugs, and treatment facilities). The list of health facilities providing DOTS and sputum smear-microscopy services during 2003–2012 was obtained from the Sidama Zone Health Department reports and database. We also cross-checked the information about health facility type, year of establishment, and availability of TB control services from the list of health facilities that provided DOTS and sputum smear microscopy services in the Sidama Zone. To ensure data quality, the principal investigator and supervisors closely supervised the data collection and data entry activities for consistency and completeness of information throughout the study period. The data were double entered, and the geographic information for DOTS and acid fast bacilli (AFB) microscopy services was downloaded using DNR Garmin 5.4.1 (2001 Minnesota) and exported to ArcGIS 10.2. We used a geographic projection of the World Geodetic System 1984, Universal Transverse Mercator Zone 37°N. We extracted the elevation (altitude) of each kebele of the Sidama Zone from ASTER Global Digital Elevation Model Version 2 (29).

### Variables and operational definitions

#### Availability

The availability of basic supplies for the TB control program (trained staff, TB control unit, laboratory service for sputum smear microscopy [microscopy and reagents], anti TB drugs, others such as availability of water and electric power supplies).

#### Physical accessibility

Distance from the census EL to the nearest health facility (DOTS, microscopy service).

#### Health service coverage

The number of health centres (a primary healthcare unit) divided by the catchment population of a given year. The Ethiopian Ministry of Health recommends that one health centre serve a population of 25,000; a clinic (former health station) was expected to serve 10,000 people. However, since 2011, the new health management information system of Ethiopia estimates primary healthcare coverage based on ratios of primary healthcare units (health centre and health post) to population (15).

#### Health extension workers

Female community health workers who are high school graduates, recruited from the local communities, trained for 1 year, salaried by the government, and working in rural communities.

### Data analysis and mapping

In 2011–2012, there was a substantial expansion of health facilities and a community-based active case-finding intervention (19, 20). Thus, we carried out the analysis for the periods 2003–2010 and 2011–2012 in addition to 2003–2012 to look for differences in CNRs.

We used 5,403 ELs, which are the most detailed data available, as inputs to measure proximity to health facilities. We computed the Euclidean distance from the EL to the nearest DOTS and TB diagnostic services using the near function of analysis tools in ArcGIS 10.2. Data on the health facilities, area, and population size of each kebele and the geographic coordinates of the EL and health facilities were linked to ArcGIS 10.2 and a base map. Data on the distance to the nearest health facility from the ELs were exported to IBM SPSS Statistics 20 and the mean distance from the nearest health facility for each kebele was computed. The proportion of locations within a varying distance from DOTS and AFB microscopy facilities (diagnostic facilities) was computed for a comparison of physical accessibility within the study area. We considered locations >10 km from the nearest health facility as areas with poor physical accessibility. The HSC was estimated based on the type of primary healthcare facility and the size of population it served, that is one health centre was expected to serve 25,000 people, one clinic was expected to serve 10,000 people, and a health post was expected to serve 5,000 people on average.

We collected data on smear-positive PTB cases from unit TB registers from all health facilities providing DOTS and computed the CNRs and treatment success (treatment completed or cured) of smear-positive PTB cases for each year (20). We used the number of smear-positive PTB cases as the numerator and the population of each district and kebele as the denominator. We carried out a linear regression analysis to look for the relationship of distance from TB control facilities (accessibility), elevation above sea level (altitude), and the population density per square kilometre with smear-positive PTB CNRs and treatment success. We assessed the associations of independent variables (accessibility, altitude, and population density) with the CNRs and treatment success using Pearson's correlation coefficients. Variables associated with the CNRs at *p*<0.2 were included in the multivariate regression model and *p*<0.05 was considered to be statistically significant.

### Ethical consideration

We obtained ethical approval from the Regional Health Bureau of southern Ethiopia. Informed consent was obtained from healthcare workers for the interview component and for geolocating the health facilities. Personal identifiers of TB cases were coded prior to analysis and medical records were kept in a secure place to help maintain the confidentiality of the clinical information of cases.

## Results

### Availability of TB control facilities

**Fig. 1:**
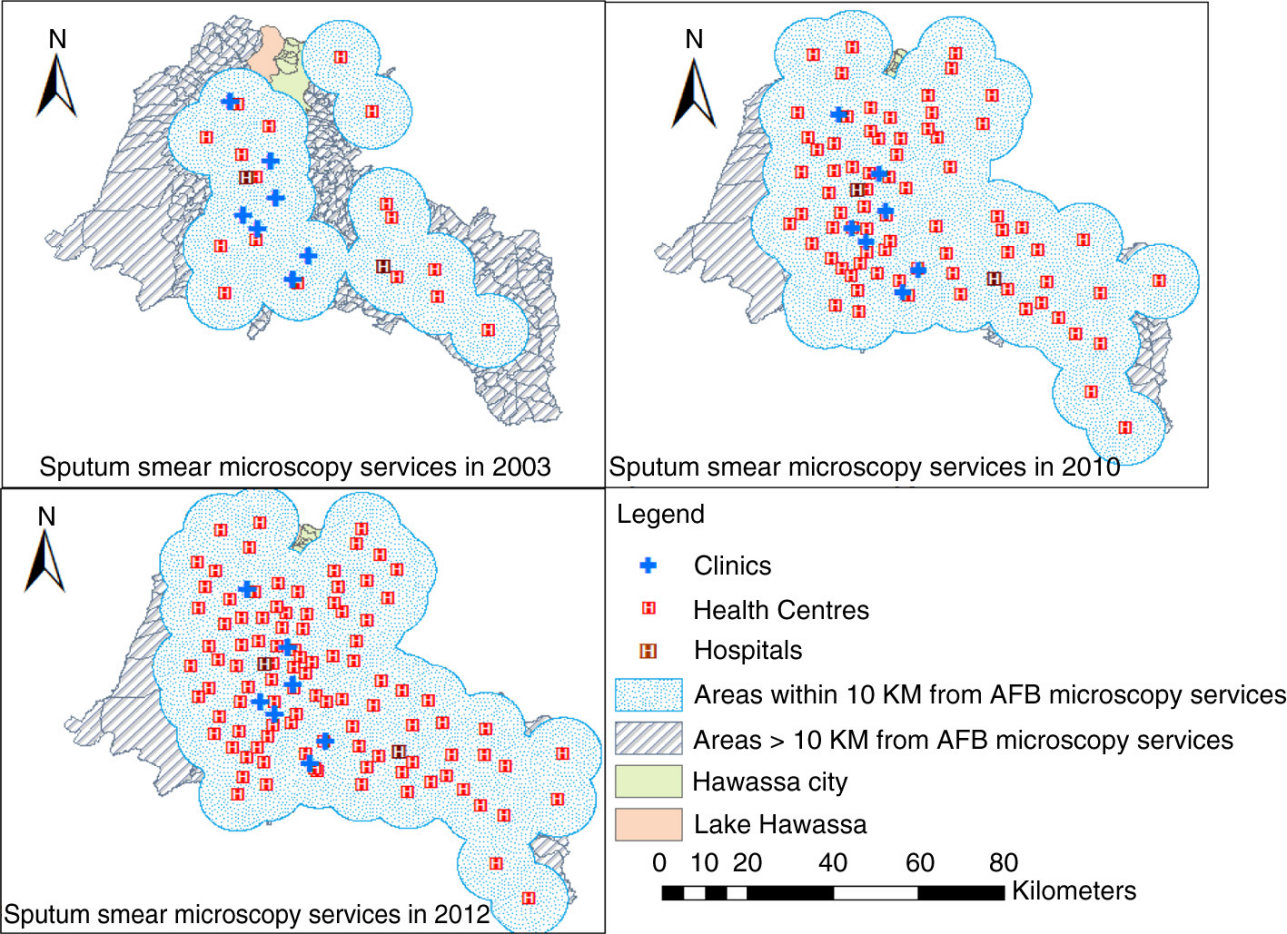
Geographic distribution of AFB microscopy services and areas within 10 km distance from the nearest TB diagnostic (AFB microscopy) facilities in the Sidama Zone, in 2003, 2010 and 2012.

**Fig. 2:**
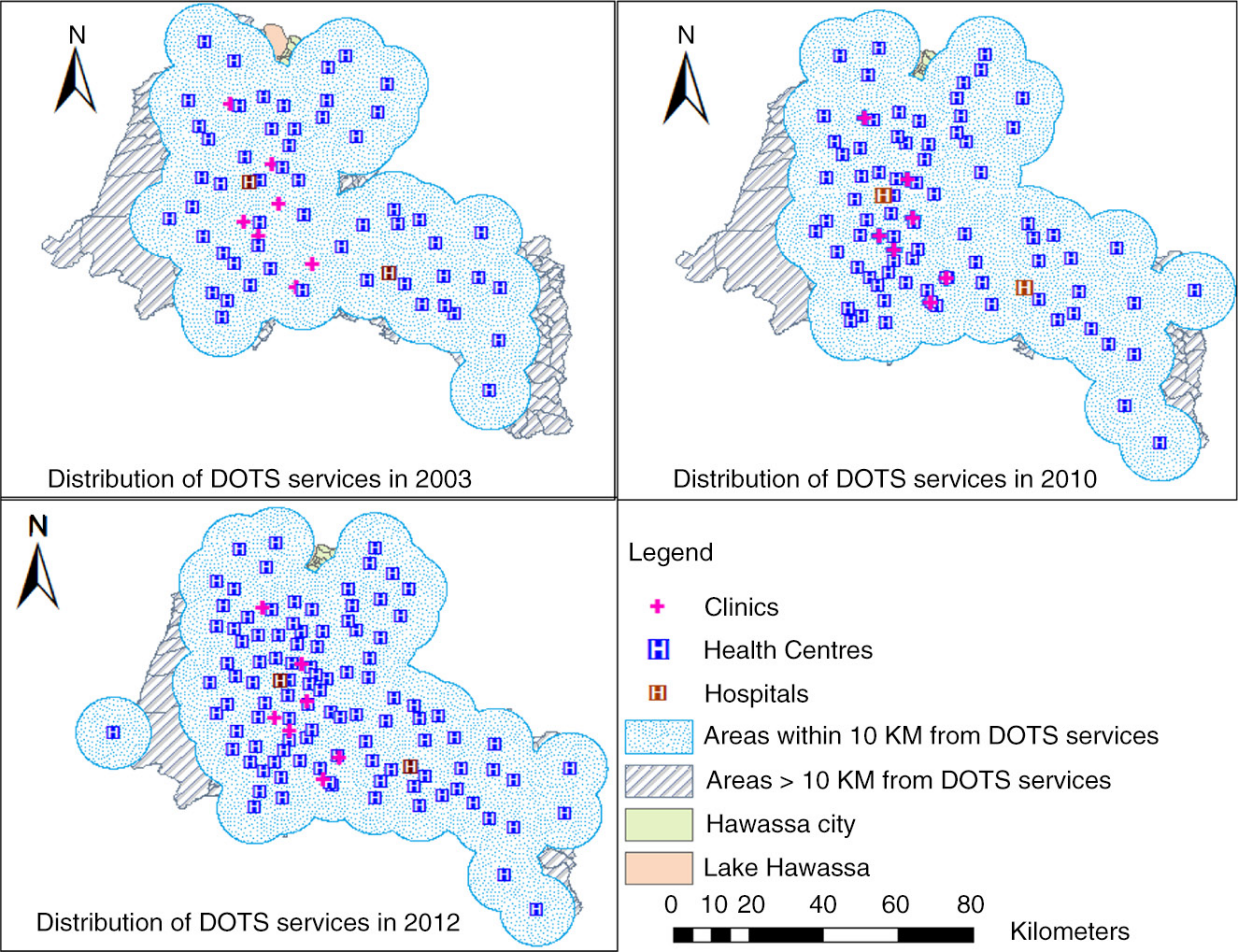
Geographic distribution of DOTs services and areas within 10 km distance from the nearest TB treatment facilities in the Sidama Zone in 2003, 2010 and 2012.

A total of 107 public and 7 non-governmental organisation health facilities were providing DOTS (Figs. 1 and 2; Supplementary Table 1). One-hundred and three (90%) healthcare providers working in TB care units were trained on TB diagnosis and treatment guidelines in the past 2 years (in 2011–2012). Ninety-one (80%) of the health facilities had the necessary reagents and supplies for sputum examination. However, 40 (35%) of the facilities did not have electric power for basic functions (Supplementary Table 1). Of 108 health facilities with sputum microscopy services, only 81 (71%) facilities were providing AFB services during the survey and the number of functional smear microscopies was 2.3 for 100,000 people (Supplementary Table 2). None of the health facilities had culture, fluorescent light-emitting diode (LED) microscopy, or automated nucleic amplification assay (Xpert MTB/RIF) services for diagnosis of TB during the study period.

### Distribution of TB service coverage; physical accessibility and smear-positive PTB CNRs and treatment outcomes

The TB control service coverage (TBSC) increased over the past 10 years by 36% (increasing from 37% in 2003 to 73% in 2012), while the variations in the coverage between districts declined (Supplementary Tables 2 and 3). In 2012, the TBSC was 73% with a ratio of one health centre to 34,068 people. The CNRs increased to 117 per 100,000 during the active case-finding intervention period (2011–2012) from 63 per 100,000 people in the prior period (2003–2010). A considerable increase in CNRs was observed in rural areas and among age groups above 35 years. Treatment outcomes (treatment success and loss to follow-up) were also improved during the active case-finding period (Table 1).

**Table 1:** Characteristics of smear-positive pulmonary tuberculosis cases during the active case-finding intervention (2011–2012) and prior period (2003–2010) in the Sidama Zone, southern Ethiopia

	Prior to active case-finding period, 2003–2010		During active case-finding intervention, 2011–2012	
		
Characteristics of subjects	N (%)	CNRs/10^5^ people	N (%)	CNRs/10^5^ people
All cases	14,630 (65)	63	7,907 (35)	117
Gender				
Men	8,113 (55.5)	70	4,122 (52)	122
Women	6,508 (45.5)	57	3,785 (48)	114
Age				
0–14	1,304 (8.9)	11	552 (8.2)	19
15–24	4,834 (33)	113	2,182 (27.6)	174
25–34	4,557 (31)	149	2,404 (30.4)	267
35–44	1,821 (12.4)	99	1,190 (15.1)	222
45–54	1,127 (7.7)	104	877 (11.1)	277
55–64	542 (3.7)	93	379 (4.8)	223
65+	309 (2.1)	54	216 (2.7)	130
Residence				
Urban	1,879 (12.8)	148	569 (7.2)	144
Rural	12,751 (87.2)	59	7,338 (92.8)	116
Treatment outcomes				
Treatment success (cured or completed)	11,284 (77)	NA	7,262 (92)	NA
Died	467 (3.2)	NA	168 (2.1)	NA
Lost to follow-up	1,742 (12)	NA	192 (2.4)	NA
Transferred	417 (2.9)	NA	114 (1.4)	NA
Treatment failure	56 (0.4)	NA	21 (0.3)	NA
Unevaluated cases	664 (4.5)	NA	150 (1.9)	NA

CNRs, case notification rates; NA, not applicable.

**Fig. 3:**
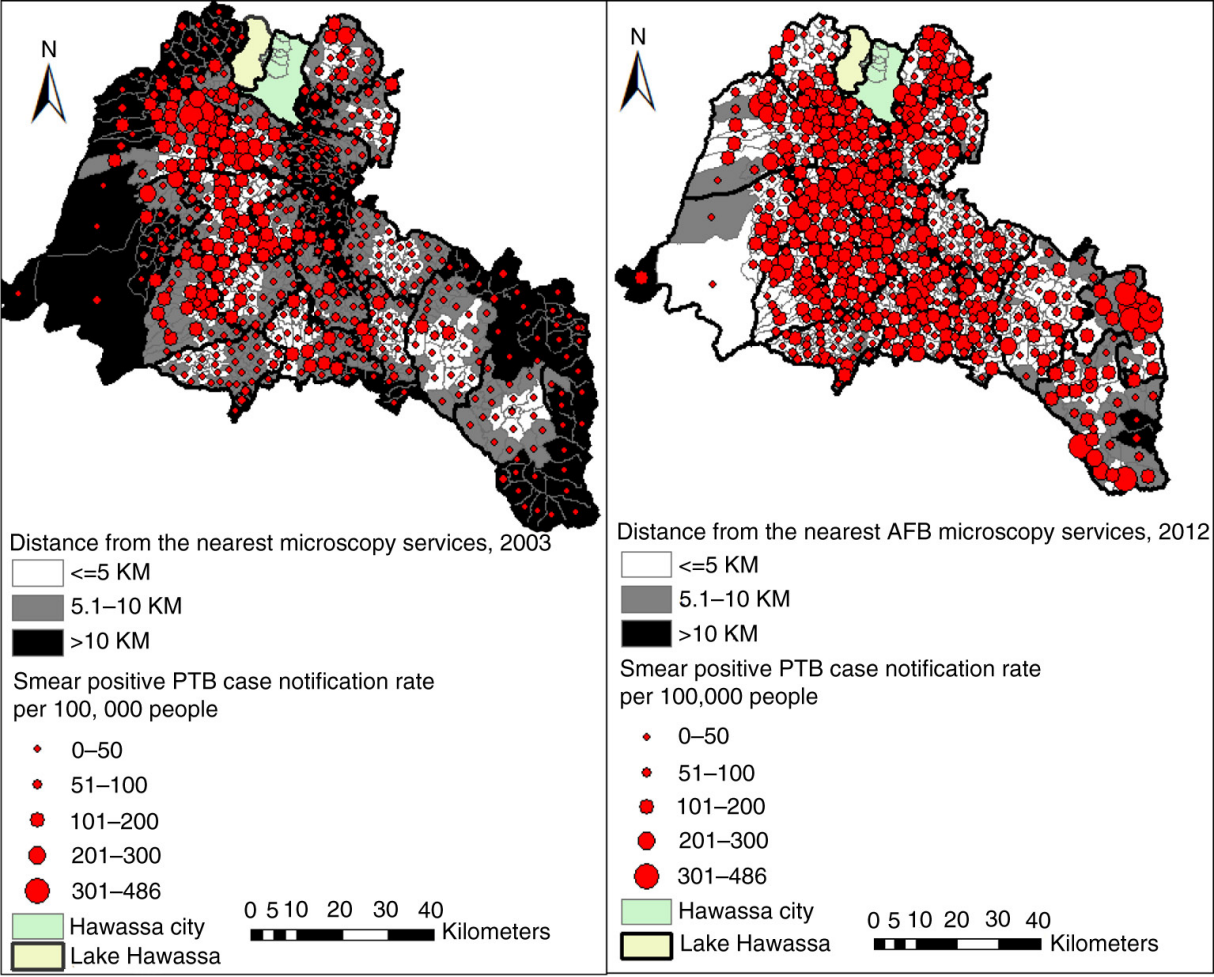
Distribution of distance from the nearest AFB facility (sputum microscopy service) and smear positive PTB case notification rates in the Sidama Zone, 2003 and 2012.

**Fig. 4:**
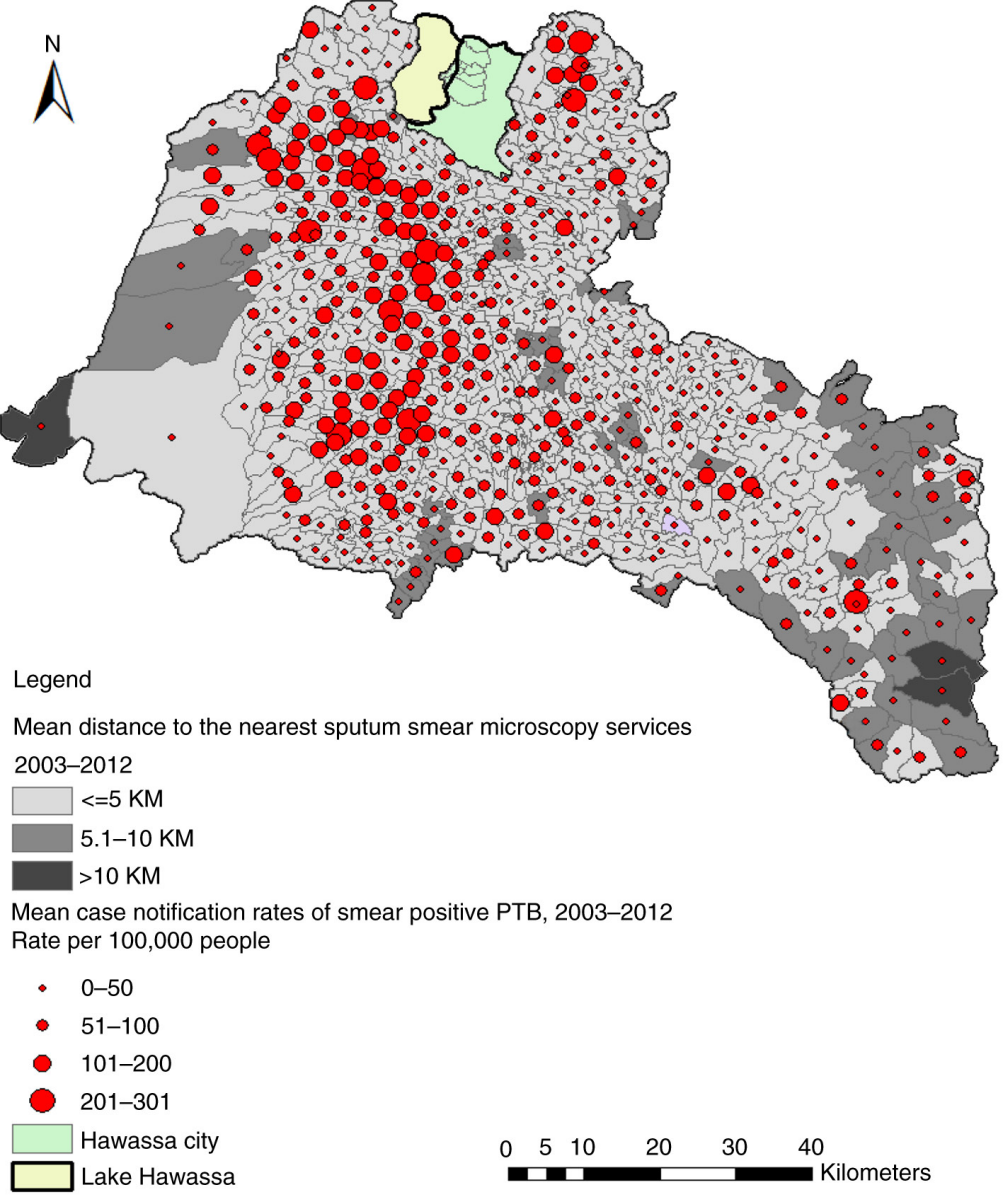
Areas within different distances from AFB (sputum smear microscopy) services and mean case notification rates of smear-positive PTB in the Sidama Zone, 2003–2012.

We found low CNRs in areas with poor physical accessibility (more than 10 km distant from the AFB facilities). However, some areas with better physical accessibility had low CNRs (Figs. 3 and 4). The proportion of locations within 10 km from the nearest AFB service increased from 39% in 2003 to 99% in 2012. Only 13% of the residential locations were within 10 km of the hospitals. The mean distance from the nearest smear microscopy unit was 7.6 km in 2003 and varied between kebeles (ranging from 1.8 to 25.5 km) and declined to 3.2 km in 2012 (ranging from 1.5 to 12.4 km). Generally, residential locations in the north-western, southern, and south-eastern borders of the study area had poor physical access and a lower HSC (Fig. 3). Altitudes within the study area range from 1,179 to 3,211 m. The proportions of DOTS services were 41% in the higher altitude (≥2,000 metres) areas, and 59% in the lower altitude areas (<2,000 metres). The mean CNRs in the lower altitude rural areas ranged from 8 to 263 per 100,000 people, while the CNRs in the higher altitude rural areas ranged from 3 to 176 per 100,000 people.

### Relationship of smear-positive PTB case reports and treatment outcomes with access to TB control services and environmental variables

In the univariate analysis, the distance to TB control services (*r*=−0.29, *p*<0.001) and altitude (*r*=−0.34, *p*<0.001) were inversely associated with smear-positive PTB CNRs. Population density was also associated with CNRs (*r*=0.22, *p*<0.001). In the multivariate linear regression model, shorter distance from TB control facilities was associated with higher CNRs (b-estimate=−0.25, *p*<0.001). This implies that for every 1 km increase in mean distance from the nearest TB diagnostic facility, the CNR of smear-positive PTB decreases by an average of 0.25 per 100,000 people. Moreover, as altitude increased (b-estimate=−0.31, *p*<0.001) the CNRs of TB decreased, whereas increase in population density (b-estimate=0.21, *p*<0.001) was associated with an increase in CNRs (Table 2). The final model was significant, with the *R*-square goodness of fit test=0.24 and the adjusted *R*-square=0.23 at *p*<0.001. Likewise, distance to TB control facilities (b-estimate=−0.27, *p*<0.001) and altitude (b-estimate=−0.30, *p*<0.001) were inversely associated with treatment success (Table 3).

**Table 2:** Multiple linear regression model of kebele level estimates for the relationship between the case notification rates of TB and accessibility, altitude, and population density in the Sidama Zone in southern Ethiopia, 2003–2012

Variables	Beta	Standard error	*t*	*p*	Variance inflation
Distance (accessibility to TB control facility)	−0.25	0.64	−6.0	<0.001	1.2
Altitude	−0.28	0.01	−7.3	<0.001	1.03
Population density	0.21	0.01	5.02	<0.001	1.19

Analysis was done using aggregated data at kebele level (*n*=563 kebeles), *R*-square=0.24, *p*-value for the model <0.001.

**Table 3:** Multiple linear regression model for the relationship between proportion of patients with treatment success (completed or cured) and accessibility, altitude, and population density in the Sidama Zone in southern Ethiopia, 2003–2012

Variables and time periods	Beta	Standard error	*t*	*p*	Variance inflation
Distance (accessibility to TB control facility)	−0.27	0.32	−6.39	<0.001	1.2
Altitude	−0.30	0.002	−7.60	<0.001	1.03
Population density	0.024	0.001	−0.56	0.579	1.2

Analysis was done using aggregated data at kebele level (*n*=563 kebeles), *R*-square=0.17, *p*-value for the model <0.001.

## Discussion

Our findings indicate that access to TB control services improved. The CNRs also increased, and the improved access and active case-finding intervention have played a role in the observed increase in TB CNRs.

In our data, distance from health facilities and altitude exhibited an inverse relationship with TB case notifications. The inverse relationship between distance and the CNRs could partly explain that areas farther away from TB control facilities have poor access or could not use existing services due to the distance barrier, which could contribute to lower case notification. Evidence shows that physical distance is one of the factors that affect utilisation of different health services (8, 30–34). Studies report various factors such as socio-economics, health seeking behaviour, individuals’ preference for service, service quality, affordability or indirect costs (35, 36), stigma, and low level of awareness about a disease that could affect the utilisation of existing facilities. On the other hand, TB CNRs depend on variations in the burden of TB in different geographic areas and the association between distance and the CNRs could be masked by differences in the burden and transmission of TB.

The performance of the TB control programme could have been influenced by low coverage or supply of microscopy and basic facilities. In our data, the number of facilities offering smear microscopy for 100,000 people was 2.3, consistent with the national report (16). However, there were variations in availability and supply of TB control facilities within the study area. Services to help improve TB diagnosis such as LED microscopy, Xpert MTB/RIF, and culture are suggested to improve TB case detection (16); however, no health facility offered these services in the study area. Improving the availability of and access to DOTS and diagnostic facilities in areas with poor access could increase the CNRs and improve treatment outcomes, which would consequently reduce infectious cases.

We found low CNRs of TB in areas with high altitude. These areas are the highlands of Sidama (2000–3211 m above sea level). They have poor access to roads and unfavourable socio-economic conditions, which could contribute to poor utilisation of the services and low disease case notification. Studies from other countries report the relationship between altitude and TB incidence (21, 24) and suggest that the oxygen pressure in different altitudes may affect or favour the proliferation and survival of *Mycobacterium* (37, 38), which might contribute to low CNRs or a lower disease burden in areas with high altitudes. Poor access to TB control facilities could also contribute to low CNRs; nonetheless, we found a significant association between altitude and TB CNRs after adjusting for distance and for population density. Follow-up studies are suggested, including other confounding factors such as socio-economic variables and biological factors to better understand the relationship between altitude and TB incidence.

Comparing the active case-finding period (2011–2012) with the prior period, the CNRs improved; however, areas with poor accessibility to health facilities had low TB CNRs. This could partly explain how poor access to diagnostic facilities might contribute to low CNRs despite the active case-finding intervention. We also found an increased CNR among older age groups and improved treatment outcomes during the active case-finding period. These data imply that improved accessibility and the active case-finding approach detected more cases among older age groups and contributed to an increase in CNRs.

Treatment success was also associated with an improved accessibility to TB control facilities during the study period (2003–2012). The proportion of treatment success was the highest in 2011–2012, which could be partly explained by a new community-based approach for enhanced case finding and treatment outcome (19). The community-based intervention decentralised the treatment to the community and improved access to TB control services (19); it could possibly have addressed other non-spatial factors that affected the utilisation of services. Non-spatial factors such as age, gender, individuals’ and providers’ perceptions, quality of care, and drug side effects could determine treatment success among TB cases (39).

Moreover, the active case-finding intervention contributed to a considerable increase in the CNRs in rural areas. This is because the intervention increased access to TB care for the rural community and increased awareness about TB, which could help early diagnosis and treatment of the disease (19). The study area was a predominantly rural setting where sociocultural factors (acceptability) could have influenced the use of existing health services. However, in the study area the provision of modern health services stretched back six decades, and the health extension workers who were recruited from the community and working in rural settings might have improved utilisation of modern health services and addressed the sociocultural barriers to seeking TB treatment.

The findings of our study could help policy- and decision makers to understand the variations in access to TB control services and their relationship with TB CNRs and treatment outcomes, which could help improve TB control programme performance. Measuring physical accessibility using GIS could help assess the distribution of and access to general healthcare delivery.

The limitations of our study were that the method we used assumed equal access to TB control facilities for the population in census ELs. The intervention that took place in 2011–2012 could have affected the results of the relationship of physical accessibility with TB CNRs and treatment outcomes. There could have been recall bias on the consistent and uninterrupted availability of drugs and TB diagnostic facilities for the period 2003–2011, although we interviewed the health personnel at health facilities for the availability of TB control facilities as well as obtaining a list of health services that provided DOTS and AFB services for each year from 2003 to 2012. We could not include socio-economic variables in the model since the data were not available at the kebele level. However, the study generated valuable information from the available data to assess physical accessibility in relation to TB service performance.

The strength of our study is that the study provides GIS-based evidence to the health system of Ethiopia using the TB program as a proxy indicator to assess physical accessibility, and the study covered a wider geographic area using ELs to measure distance from the health facilities.

## Conclusions

Accessibility to TB control services improved despite geographic variations. Moreover, physical access and altitude were associated with TB CNRs and treatment outcomes, and the CNRs were higher in areas where people had better access to TB diagnostic and treatment centres. The community-based intervention also played an important role in the increased case notification and treatment outcomes. Efforts need to be made to improve access to TB control facilities in areas characterised by poor accessibility to services and in areas with lower CNRs, so as to improve control of TB.

## Supplementary Material

Accessibility to tuberculosis control services and tuberculosis programme performance in southern Ethiopia

Accessibility to tuberculosis control services and tuberculosis programme performance in southern Ethiopia

## References

[1] Tanahashi T (1978). Health service coverage and its evaluation. Bull World Health Organ.

[2] Penchansky R, Thomas JW (1981). The concept of access: definition and relationship to consumer satisfaction. Med Care.

[3] Higgs G (2004). A literature review of the use of GIS-based measures of access to health care services. Health Serv Outcomes Res Methodol.

[4] O'Connell T, Rasanathan K, Chopra M (2014). What does universal health coverage mean?. Lancet.

[5] Joseph AE, Phillips DR (1984). Accessibility and utilization; geographical perspectives on health care delivery.

[6] Christie S, Fone D (2003). Equity of access to tertiary hospitals in Wales: a travel time analysis. J Public Health Med.

[7] Brabyn L, Skelly C (2002). Modeling population access to New Zealand public hospitals. Int J Health Geogr.

[8] Jordan H, Roderick P, Martin D, Barnett S (2004). Distance, rurality and the need for care: access to health services in South West England. Int J Health Geogr.

[9] Brabyn L, Barnett R (2004). Population need and geographical access to general practitioners in rural New Zealand. N Z Med J.

[10] Jacoby I (1991). Geographic distribution of physician manpower: the GMENAC (Graduate Medical Education National Advisory Committee) legacy. J Rural Health.

[11] Cromley EK, McLafferty SL (2012). GIS and public health.

[12] Tanser F, Wilkinson D (1999). Spatial implications of the tuberculosis DOTS strategy in rural South Africa: a novel application of geographical information system and global positioning system technologies. Trop Med Int Health.

[13] Wilkinson D, Tanser F (1999). GIS/GPS to document increased access to community-based treatment for tuberculosis in Africa. Geographic information system/global positioning system. Lancet.

[14] FMOH (2010). Health Sector Development Programme IV 2010/11 – 2014/15.

[15] FMOH (2014). HMIS indicator definitions: technical standards.

[16] WHO (2014). Global tuberculosis report 2014.

[17] FMOH (2013/14). Annual performance report.

[18] Datiko DG, Lindtjorn B (2009). Health extension workers improve tuberculosis case detection and treatment success in southern Ethiopia: a community randomized trial. PLoS One.

[19] Yassin MA, Datiko DG, Tulloch O, Markos P, Aschalew M, Shargie EB (2013). Innovative community-based approaches doubled tuberculosis case notification and improve treatment outcome in Southern Ethiopia. PLoS One.

[20] Dangisso MH, Datiko DG, Lindtjorn B (2014). Trends of tuberculosis case notification and treatment outcomes in the sidama zone, southern Ethiopia: ten-year retrospective trend analysis in urban-rural settings. PLoS One.

[21] Tanrikulu AC, Acemoglu H, Palanci Y, Dagli CE (2008). Tuberculosis in Turkey: high altitude and other socio-economic risk factors. Public Health.

[22] Liu JJ, Yao HY, Liu EY (2005). Analysis of factors affecting the epidemiology of tuberculosis in China. Int J Tuberc Lung Dis.

[23] Couceiro L, Santana P, Nunes C (2011). Pulmonary tuberculosis and risk factors in Portugal: a spatial analysis. Int J Tuberc Lung Dis.

[24] Vargas MH, Furuya ME, Perez-Guzman C (2004). Effect of altitude on the frequency of pulmonary tuberculosis. Int J Tuberc Lung Dis.

[25] Vree M, Hoa NB, Sy DN, Co NV, Cobelens FG, Borgdorff MW (2007). Low tuberculosis notification in mountainous Vietnam is not due to low case detection: a cross-sectional survey. BMC Infect Dis.

[26] Tadesse T, Demissie M, Berhane Y, Kebede Y, Abebe M (2013). Long distance travelling and financial burdens discourage tuberculosis DOTs treatment initiation and compliance in Ethiopia: a qualitative study. BMC Public Health.

[27] Castelnuovo B (2010). A review of compliance to anti tuberculosis treatment and risk factors for defaulting treatment in Sub Saharan Africa. Afr Health Sci.

[28] CSA (2012). Population.

[29] USEROS (2011). ASTER global digital elevation model version 2.

[30] Malqvist M, Sohel N, Do TT, Eriksson L, Persson LA (2010). Distance decay in delivery care utilisation associated with neonatal mortality. A case referent study in northern Vietnam. BMC Public Health.

[31] Al-Taiar A, Clark A, Longenecker JC, Whitty CJ (2010). Physical accessibility and utilization of health services in Yemen. Int J Health Geogr.

[32] Hounton S, Chapman G, Menten J, De Brouwere V, Ensor T, Sombie I (2008). Accessibility and utilisation of delivery care within a skilled care initiative in rural Burkina Faso. Trop Med Int Health.

[33] Acharya AB, Nyirenda JC, Higgs GB, Bloomfield MS, Cruz-Flores S, Connor LT (2011). Distance from home to hospital and thrombolytic utilization for acute ischemic stroke. J Stroke Cerebrovasc Dis.

[34] Ayeni B, Rushton G, McNulty ML (1987). Improving the geographical accessibility of health care in rural areas: a Nigerian case study. Soc Sci Med.

[35] Ford CM, Bayer AM, Gilman RH, Onifade D, Acosta C, Cabrera L (2009). Factors associated with delayed tuberculosis test-seeking behavior in the Peruvian Amazon. Am J Trop Med Hyg.

[36] Rosenstock IM (1996). Why people use health services. Milbank Mem Fund Q.

[37] Chadha VK (2005). Tuberculosis epidemiology in India: a review. Int J Tuberc Lung Dis.

[38] Sever JL, Youmans GP (1957). The relation of oxygen tension to virulence of tubercle bacilli and to acquired resistance in tuberculosis. J Infec Dis.

[39] Muture BN, Keraka MN, Kimuu PK, Kabiru EW, Ombeka VO, Oguya F (2011). Factors associated with default from treatment among tuberculosis patients in Nairobi province, Kenya: a case control study. BMC Public Health.

