# Supplementary material for: Accessibility to tuberculosis control services and tuberculosis programme performance in southern Ethiopia
Source: Glob Health Action. 2015 Nov 20;8:10.3402/gha.v8.29443. doi: 10.3402/gha.v8.29443 (PMC4655224; doi:10.3402/gha.v8.29443)
Supplement: Accessibility to tuberculosis control services and tuberculosis programme performance in southern Ethiopia [file GHA-8-29443-s001.docx]

Supplementary 1 Table. Proportions of residential locations with varying distance to microscopy services for tuberculosis diagnosis in the Sidama Zone in southern Ethiopia, 2003, 2010 and 2012

| Distance to Microscopy services | Year | | |
| --- | --- | --- | --- |
|  | 2003 | 2010 | 2012 |
|  | Proportion of enumeration locations  N (%) | Proportion of enumeration locations  N (%) | Proportion of enumeration locations  N (%) |
| <1 km | 94 (2) | 296 (6) | 402 (7) |
| 1.1-5 km | 1,855 (34) | 3,624 (67) | 4,294 (79) |
| 5.1-10 km | 2091 (39) | 1,361 (25) | 680 (13) |
| 10.1-15 km | 293 (6) | 113 (2) | 26 (1) |
| 15.1-20 km | 319 (6) | 8 (0.1) | 0 |
| >20 km | 121 (2) | 1 | 1 |
