# Supplementary material for: Accessibility to tuberculosis control services and tuberculosis programme performance in southern Ethiopia
Source: Glob Health Action. 2015 Nov 20;8:10.3402/gha.v8.29443. doi: 10.3402/gha.v8.29443 (PMC4655224; doi:10.3402/gha.v8.29443)
Supplement: Accessibility to tuberculosis control services and tuberculosis programme performance in southern Ethiopia [file GHA-8-29443-s002.docx]

Supplementary information

S1Table: Availability and distribution of TB control facilities by districts in the Sidama Zone in southern Ethiopia, 2003-2012

| Districts | Number of  DOTS facilities | Number of functional microscopy for AFB | Source of power | | | | Facilities with functional water supply | |
| --- | --- | --- | --- | --- | --- | --- | --- | --- |
|  |  |  | Electricity | Generator | Solar | | protected water source | Others* |
| Shebedino | 8 | 6 | 6 | 1 | | - | 3 | 4 |
| Hawassa Zuriya | 3 | 3 | 2 | - | | - | 2 | 1 |
| Arbegona | 6 | 3 | 2 | - | | - | 1 | 3 |
| Dale | 9 | 8 | 6 | - | | - | 3 | 5 |
| Aleta Wondo | 8 | 5 | 5 | - | | - | 3 | 4 |
| Dara | 5 | 3 | 3 | - | | - | 2 | 1 |
| Hula | 7 | 5 | 4 | - | | - | 3 | 1 |
| Bensa | 7 | 6 | 1 | 2 | | 2 | 3 | 3 |
| Aroresa | 4 | 3 | 2 | 1 | | 1 | - | 4 |
| Boricha | 10 | 8 | 6 | - | | - | 2 | 7 |
| Gorche | 5 | 4 | 1 | - | | 1 | - | 1 |
| Malga | 4 | 3 | 2 | - | | - | 3 | 1 |
| Wonsho | 5 | 3 | 2 | - | | - | 1 | 2 |
| Loka Abaya | 7 | 3 | 2 | 1 | | 2 | - | 6 |
| Chire | 3 | 2 | 0 | - | | 2 | 1 | 1 |
| Bursa | 4 | 2 | 2 | 1 | | - | 2 | 2 |
| Chuko | 7 | 5 | 4 | 1 | | - | 5 | 2 |
| Bona | 5 | 3 | 3 | - | | - | 2 | 3 |
| Wondo Genet | 4 | 3 | 3 | - | | - | 1 | 1 |
| AletaWondo town | 1 | 1 | 1 | - | | - | 1 | - |
| Yirgalem town | 2 | 2 | 2 | - | | - | 2 | - |
| Total | 114 | 81 | 59 | 7 | | 8 | 40 | 48 |

Others* = collecting rain water during rainy season or water from other sources outside health facilities

S2 Table: Trends of TB control service expansion and smear positive PTB CNRs in the Sidama Zone in southern Ethiopia, 2003-2012

| Year | Number of DOTS facilities | DOTS service ratio per 10^5^ people | Number Functional microscopy services | Functional microscopy ratio per 10^5^ people | Health service coverage (%) | PTB+ CNR  Per 10^5^ people | Treatment success (Completed or Cured %) | Lost-to-follow up (Defaulted %) |
| --- | --- | --- | --- | --- | --- | --- | --- | --- |
| 2003 | 65 | 2.7 | 26 | 1.1 | 37 | 55 | 85 | 8.2 |
| 2004 | 67 | 2.6 | 29 | 1.1 | 38 | 62 | 80 | 10.2 |
| 2005 | 67 | 2.5 | 29 | 1.1 | 36 | 58 | 76 | 6.4 |
| 2006 | 67 | 2.4 | 31 | 1.1 | 36 | 51 | 76 | 11.1 |
| 2007 | 68 | 2.3 | 34 | 1.1 | 37 | 58 | 82 | 10.7 |
| 2008 | 70 | 2.3 | 38 | 1.2 | 38 | 82 | 78 | 14.2 |
| 2009 | 74 | 2.4 | 46 | 1.5 | 44 | 73 | 74 | 13.2 |
| 2010 | 86 | 2.7 | 76 | 2.4 | 75 | 67 | 71 | 17.3 |
| 2011 | 112 | 3.4 | 80 | 2.4 | 72 | 122 | 92 | 3.2 |
| 2012 | 114 | 3.3 | 81 | 2.3 | 73 | 111 | 93 | 1.7 |

PTB+ = smear-positive pulmonary tuberculosis

Table 3: Health service coverage (health facility to population ratios) and smear positive TB case notification rates by districts in the Sidama zone, 2003 and 2012

| Districts | 2003 |  |  | | 2012 |  |  |
| --- | --- | --- | --- | --- | --- | --- | --- |
|  | Number of  health (DOTS) facilities | Health facility to population ratio (Coverage %) | | PTB+ CNRs | Number of  health (DOTS) facilities | Health facility to population ratio (Coverage %) | PTB+ CNRs |
| Shebedino | 6 | 42 | | 89 | 8 | 74 | 128 |
| Hawassa Zuriya | 2 | 25 | | 61 | 3 | 52 | 94 |
| Arbegona | 4 | 56 | | 7 | 6 | 80 | 57 |
| Dale | 5 | 41 | | 94 | 9 | 76 | 159 |
| AletaWondo | 5 | 34 | | 53 | 8 | 78 | 122 |
| Dara | 5 | 58 | | 9 | 5 | 68 | 57 |
| Hula | 3 | 48 | | 49 | 7 | 84 | 111 |
| Bensa | 7 | 45 | | 27 | 7 | 61 | 84 |
| Aroresa | 1 | 26 | | 4 | 4 | 51 | 130 |
| Boricha | 5 | 43 | | 122 | 10 | 69 | 102 |
| Gorche | 2 | 23 | | 25 | 5 | 103 | 77 |
| Malga | 3 | 58 | | 32 | 4 | 99 | 75 |
| Wonsho | 1 | 13 | | 54 | 5 | 121 | 136 |
| Loka Abaya | 3 | 36 | | 58 | 7 | 132 | 110 |
| Chire | 1 | 10 | | 0 | 3 | 54 | 229 |
| Bursa | 1 | 12 | | 41 | 4 | 84 | 91 |
| Chuko | 3 | 36 | | 94 | 7 | 91 | 117 |
| Bona | 4 | 64 | | 23 | 5 | 73 | 80 |
| Wondo Genet | 2 | 29 | | 75 | 4 | 70 | 208 |
| Aleta Wondo town | 1 | 143 | | 57 | 1 | 90 | 140 |
| Yirgalem town | 1 | 104 | | 249 | 2 | 65 | 180 |
| Total | 65 | 37 | | 55 | 114 | 73 | 111 |

PTB+ = smear positive pulmonary tuberculosis

CNRs = case notification rates
